# The role of complete blood inflammation markers in the prediction of spontaneous abortion

**DOI:** 10.12669/pjms.346.15939

**Published:** 2018

**Authors:** Funda Yildirim Bas, Esra Nur Tola, Suheyla Sak, Basak Asli Cankaya

**Affiliations:** 1 *Dr. Funda Yildirim Bas, Department of Medicine, Suleyman Demirel University, Isparta, Turkey*; 2 *Dr. Esra Nur Tola, Department of Medicine, Suleyman Demirel University, Isparta, Turkey*; 3 *Dr. Suheyla Sak, Department of Medicine, Suleyman Demirel University, Isparta, Turkey*; 4 *Dr. Basak Asli Cankaya, Department of Medicine, Suleyman Demirel University, Isparta, Turkey*

**Keywords:** Abortion, Complete blood count, Inflammation

## Abstract

**Objective:**

To investigate relationships between spontaneous abortion and complete blood count inflammation markers and their role in predicting spontaneous abortion.

**Methods:**

This study was conducted at Department of Obstetrics and Gynecology between January 2012 and January 2017. A total of 570 participants, 325 diagnosed with spontaneous abortion and 245 control patients who underwent timely births were included into our study. The complete blood count inflammation markers included white blood cell (WBC), neutrophil (N), lymphocyte (L), neutrophil-lymphocyte ratio (NLR), mean platelet volume (MPV) and platelet-lymphocyte ratio level (PLR) were recorded.

**Results:**

There was difference between the abortion groups and control groups in terms of complete blood count (CBC) inflammation markers, including WBC, PLT, neutrophil, lymphocyte, NLR, PLR, and MPV. We found decreased MPV, PLR levels and increased N, L and NLR in the first. and second. Abortion groups compared with the control group. WBC, N, L and NLR were positive predictive markers, and albeit with low sensitivity and specificity, MPV, PLR were found to be a negative predictive marker for the evaluation of spontaneous abortion

**Conclusions:**

Unlike several difficult and invasive tests, a CBC is a simple, inexpensive and easily available test. CBC inflammation markers, including WBC, N, L, NLR, PLR, and MPV, which were evaluated at the sixth gestational week, can be used for the risk assessment of spontaneous abortion in pregnancy.

## INTRODUCTION

Spontaneous abortion is defined as a clinically recognized pregnancy loss before the 20th week of gestation, or an extraction of an embryo weighing 500 g or less. It is the most common pregnancy complication and affects approximately 15% of all pregnancies.^1^

Many factors have been associated with spontaneous abortions, such as genetic disorders, chromosomal abnormalities, endocrinology imbalances, and immunologic dysfunctions.^2^ One etiopathogenetic factor, with its pathophysiology, is inflammation. There is an association between inflammation and uterine contractions with progressive cervical effacement and dilation, which are responsible for the onset and maintenance of both term and preterm labour.^3^

Some complete blood count (CBC) parameters, such as WBC (white blood cell) types changes during pregnancy with the ratio of granulocytes and T helper (Th)-1 lymphocytes being significantly elevated with a concomitant reduction in the ratio of Th-2 lymphocytes and monocytes are important.^4^ Macrophages and monocytes have an essential role in the development of the placenta. They promote the invasion of extra villous trophoblasts, spiral artery remodelling and the parturition process. However, controversy exists concerning whether the deregulation of these cells may lead to pregnancy complications, such as abortion, preeclampsia and preterm labour.^5^

CBC is an inexpensive, easy to access and commonly used test, and is recommended at the first initial stages of pregnancy to exclude pathological complications, such as anaemia, thrombocytopenia, bleeding disorder, thrombosis and thrombophilia.^6^

Measuring blood cell subtype ratios, such as WBC, neutrophil (N), lymphocyte (L), the neutrophil-lymphocyte ratio (NLR), the mean platelet volume (MPV), the platelet-lymphocyte ratio level (PLR) and red blood cell distribution width (RDW) might provide prognostic and diagnostic clues to diseases. In recent years, NLR, PLR and MPV are also increasingly used as markers of chronic inflammation.^7^ NLR, as an indicator of systemic inflammation in some diseases (preeclampsia, coronary artery disease, ulcerative colitis etc.)^8^-^10^ PLR could serve as a biological marker of both thrombosis and inflammation.^11^

The role of inflammation in the etiopathogenesis of spontaneous abortion, the easy determination of these processes by CBC, and the scarcity of studies in these fields motivated us to investigate the relationship between CBC inflammation markers and spontaneous abortion. We also determined the role of complete blood parameters in the prediction of abortion.

## METHODS

We looked at all the data of pregnant patients who admitted to University, Faculty of Medicine, Department of Obstetrics and Gynecology between January 2012 and January 2017.A total of 570 participants, 325 diagnosed with spontaneous abortion (1. trimester abortion group: (<14 w) n=173 and 2. trimester abortion group (>14 w): n=152) and 245 control patients who underwent timely births were included into our study. Control group consisted of women who had given birth at term without complications. They were matched by age and body mass index (BMI). Only normal weight (BMI<25 kg/m^2^) participants were included to our study to rule out the effect of obesity on inflammation. The definition of spontaneous abortion was considered as the exclusion of foetal joints before the 20th gestational week and less than 500 grams.^1^ Gestational age was found by measuring the crown-to-rump length with transvaginal ultrasound and at the time of presence of foetal heart rate. The CBC parameters included WBC, N, L, NLR, MPV and PLR were recorded before six gestational weeks.

Exclusion criteria for all participants were smoking and alcohol use, recurrent abortions, obesity, systemic diseases (hypertension, diabetes, etc.), malignancy, presence of infectious disease, autoimmune diseases, use of anti-inflammatory drugs or glucocorticoids, and other chronic inflammatory conditions (arthritis, etc.).

### Laboratory evaluation

Venous blood samples were taken into the ethylene diamine tetra acetic acid possessing tubes. The CBC was performed using an auto blood analyser (Cell-Dyn 3700. Abbott®, USA).

### Statistical analysis

Data were analysed with SPSS software, version 17.0 for Windows (SPSS for Windows, Chicago, IL, USA). An independent samples *t*-test and one way ANOVA was used to compare variables where appropriate. Correlations between continous variables were evaluated using Pearson’s correlation analysis or Spearman’s rank test. Logistic regression analysis was used to evaluate the association between dependent and independent variables. To estimate the sensitivity and specificity, a receiver-operator curve (ROC) analysis was performed. A *p* value < 0.05 was accepted as statistically significant.

### Ethical approval

The study was approved by Local Ethics Committee of Suleyman Demirel University with the protocol number 72867572-050-5025.

## RESULTS

A total of 570 participants, 325 diagnosed with abortion (1. trimester abortion group: (<14 w) n=173 and 2. trimester abortion group (>14 w): n=152) and 245 control patients who underwent timely births were included into the study.

### Baseline characteristics

The mean age of all participants was 30.86 ± 6.06 years. There was no significant difference between first Trimester, and second Trimester abortion groups and control group in terms of age and BMI (p=0.06, p=0.32) (Table-I). Comparison of basal demographic characteristics and complete blood count parameters between control and abortion groups is shown in Table-I.

**Table-I:** Comparison of basal demographic characteristics and complete blood count parameters between control and abortion groups.

	Control group (n=245)	Abortion (n=325)		p value
		1.trimester abortion group (n=173)	2. trimester abortion group (n=152)	
AGE (years)	30.15±5.62	31.88±6.43	30.87±6.19	0.06
BMI(kg/m^2^)	23.32±1.65	23.28±1.70	23.56±1.40	0.32
WBC(10³/µl)	8.48±2.28	7.13±3.80	8.81±4.41	<0.0001
PLT(10³/µl)	241.11±61.40	198.32±125.91	192.73±116.62	<0.0001
N(10³/µl)	5.76±1.89	14.78±14.45	14.13±12.41	<0.0001
L(10^³^/µl)	2.01±0.63	55.72±93.60	45.66±85.95	<0.0001
NLR	3.07±1.22	4.16±2.19	5.77±5.42	<0.0001
PLR	127.83±39.39	107.01±77.86	111.48±80.55	0.03
MPV(fL)	8.67±0.94	6.72±2.41	7.34±1.92	<0.0001

**BMI:** Body mass index; WBC: White blood cell; PLT: Platelet; N: neutrophil; L: lymphocyte **NLR:** Neutrophil-to-lymphocyte ratio; PLR: Platelet-to-lymphocyte ratio; MPV: Mean platelet volume.

**Fig.1:**
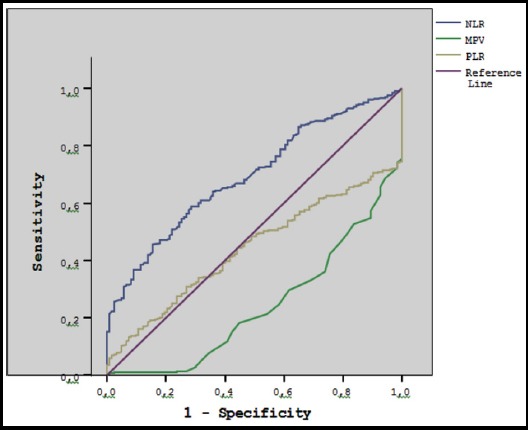
ROC curve analysis of complete blood count parameter for the determination of the diagnosis of abortion

Regression analysis of complete blood count parameter for the determination of the diagnosis of abortion (Table-II). We calculated the cut-off value of NLR, PLR and MPV in the prediction of abortion via the ROC analysis (Fig.1). For NLR, at a cut-off level of 3.13, the sensitivity was 67% and the specificity was 57%, for PLR at a cut-off level of 119.0, the sensitivity was 50% and the specificity was 47% and for MPV at a cut-off level of 7.95, the sensitivity was 46% and the specificity was 22%.

**Table-II:** Regression analysis of complete blood count parameter for the determination of the diagnosis of abortion.

	β	P value	OR (95% CI)
NLR	0.41	<0.001	1.51 (1.31-1.74)
PLR	-0.06	0.01	0.99(0.98-0.99)
MPV	-0.99	<0.001	0.37(0.28-0.48)

Covariates: PLT, N, L, NLR, PLR, MPV, NLR: Neutrophil-to-lymphocyte ratio; PLR: Platelet-to-lymphocyte ratio; MPV: Mean platelet volume.

In the logistic regression analyses, we found a positive predictive effect of NLR and MPV in the prediction of one. Trimester abortion groups details are given in Table-III. For NLR, at a cut-off level of 3.24, the sensitivity was 62% and the specificity was 50%, for MPV at a cut-off level of 7.85, the sensitivity was 50% and the specificity was 33%.

**Table-III:** Regression analysis of complete blood count parameter for the determination of the diagnosis of first trimester abortion.

	β	P value	OR (95% CI)
NLR	0.19	<0.001	1.2(1.03-1.41)
MPV	-0.92	<0.001	0.39(0.30-0.52)

Covariates: PLT, N, L, NLR, PLR, MPV. NLR: Neutrophil-to-lymphocyte ratio, MPV: Mean platelet volume.

In the logistic regression analyses, we found a positive predictive effect of NLR, PLR and MPV in the prediction of 2. Trimester abortion (Table-IV). For NLR at a cut-off level of 3. 34, the sensitivity was 68% and the specificity was 65%, for PLR, at a cut-off level of 118. 4, the sensitivity was 50% and the specificity was 46% and for MPV at a cut-off level of 8.05, the sensitivity was 40% and the specificity was 25%.

**Table-IV:** Regression analysis of complete blood count parameter for the determination of the diagnosis of second trimester abortion.

	β	P value	OR (95% CI)
NLR	0.63	<0.001	1.79 (1.45-2.21)
PLR	-0.14	<0.001	0.98(0.97-0.99)
MPV	-1.11	<0.001	0.32(0.23-0.46)

Covariates: WBC, PLT, N, L, NLR, PLR, MPV NLR: Neutrophil-to-lymphocyte ratio; PLR: Platelet-to-lymphocyte ratio; MPV: Mean platelet volume.

## DISCUSSION

In the present study, we investigated CBC inflammation markers, which were screened at the sixth week of gestation, between the first and second Trimester, Trimester spontaneous abortion groups and control patients who underwent timely births.

There was difference between the abortion groups and control groups in terms of CBC inflammation markers. We found decreased MPV, PLR levels and increased N, L and NLR in the one and two Abortion groups compared with the control group. WBC, N, L and NLR were positive predictive markers, and albeit with low sensitivity and specificity, MPV, PLR were found to be a negative predictive marker for the evaluation of spontaneous abortion. These data suggest that spontaneous abortion may be associated with inflammation. The causes of abortion are often unknown but there have been associations between abortion and chromosomal aberrations, ethnic origin, very low or very high BMI, chronic medication use, smoking and alcohol consumption and infections.^12^,^13^ Specifically, 15% of early abortions and 66% of late abortions have been attributed to infections.^14^ Infections during pregnancy may result in a variety of adverse obstetrical outcomes, including preterm delivery, preterm membrane rupture, spontaneous abortion, congenital infections and congenital abnormalities.^15^ The role of inflammation is important and necessary for successful pregnancies, however aberrant and persistent inflammation and the lack of resolution by anti-inflammatory cytokine-producing cells can lead to a variety of pregnancy disorders depending on various factors.

Systemic inflammation can be measured using a variety of biochemical and haematological markers. Recent evidence indicates that measurements of the ratio of subtypes of blood cells, like NLR, PLR, and the lymphocyte to monocyte ratio, might have prognostic significance for diseases related to inflammation.^16^ In recent years, NLR, as an indicator of systemic inflammation, has been studied in preeclampsia,^8^ coronary artery disease,^9^ ulcerative colitis.^10^ However, there have been no reports on an association between abortion and NLR. In this study we have found positive marker of NLR for the evaluation of spontaneous abortion.

PLR and MPV, which has been the indicator most commonly used as a marker in previous studies, is an important risk factor for the development of atherothrombosis and embolism. Increased MPV has been defined as an independent risk factor in the development of thromboembolism.^17^ While recent studies have found increased MPV levels in cardiovascular disease,^18^ diabetes mellitus,^19^ polycystic ovarian syndrome,^20^ hypercholesterolemia,^21^ severe anaemia^22^ obesity, hypertension and smoking^23^ low birth weight pregnancies, preeclampsia and recurrent abortions.^24^ MPV values were reported to be negatively correlated with pregnancy and implantation rate, and PLR levels were found to be positively associated with miscarriages in patients with PCOS.^11^ In present study, MPV and PLR levels were found to be a negative predictive marker for the evaluation of spontaneous abortion. We thought this result was related to our patient group, who did not have chronic diseases and recurrent abortions.

### Limitations of the study

It includes retrospective design and use of data from a single tertiary centre. This limits the inability to calculate population-based rates. Despite these limitations, this was the first study that evaluated the association between CBC inflammation markers and spontaneous abortion.

## CONCLUSION

Unlike several difficult and invasive tests, CBC is a simple, inexpensive and easily available test. CBC inflammation markers, which were evaluated at the sixth gestational week, can be used for the risk assessment of spontaneous abortion in pregnancy. However, these results must be supported by further prospective large-scale studies.

### Authors Contribution

**FYB, ENT** conceived, designed and did statistical analysis & editing of manuscript

**FYB, ENT, SS, BAC** did data collection and prepared the manuscript

**FYB** did review and final approval of manuscript.
